# Enhancing Elderly Health Through Physical Activities: Insights From a Global Bibliometric Review, 1900–2023

**DOI:** 10.1002/agm2.70069

**Published:** 2026-03-23

**Authors:** Hongman Wang, Bo Xie, Can Liu, Yifan Song

**Affiliations:** ^1^ School of Humanities Southeast University Nanjing China; ^2^ Zhongda Hospital, Medical School Southeast University Nanjing China; ^3^ School of Health Humanities Peking University Beijing China

**Keywords:** bibliometric review, elderly, physical activity, Web of Science Core Collection

## Abstract

**Objective:**

This article conducted a bibliometric analysis on research of older adults' health and physical activity (PA) to enrich the academic community's comprehension and research of this field.

**Methods:**

A total of 10,778 documents published from 1900 to 2023 were included. We used Citespace to analyze global research trends, identifying core journals, highlighting collaboration networks, and citation bursts within the field of physical activity and older adults' health.

**Results:**

The publications rise consistently, with close collaboration found among researchers, institutions, and countries. Developed countries made a dominant contribution. The recent research hotspots include healthy aging, the impact of physical activities on the mental health of older adults, and the utilization of virtual reality to enhance physical activity in old people.

**Conclusion:**

The study suggests that promoting physical activity among older adults is crucial for healthy aging. It emphasizes the role of technology in facilitating exercise participation and the importance of research from developing countries to advance global healthy aging strategies.

## Graphical abstract

Bibliometric analysis reveals rising global research on physical activity and elderly health, dominated by developed nations. Key hotspots include healthy aging, physical activity's mental health benefits, and VR exercise interventions. Promoting physical activity is vital for healthy aging, requiring greater technology integration and research from developing countries.

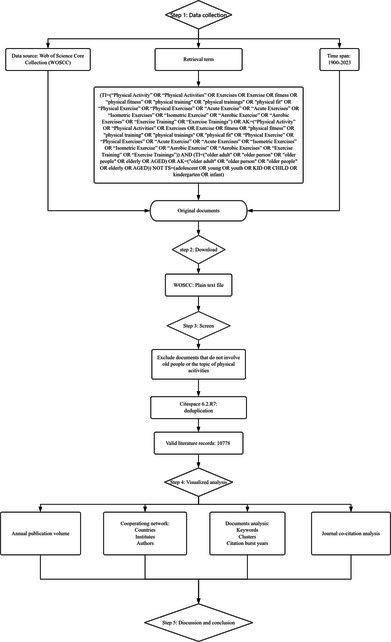

## 1 Introduction

Population aging is becoming a globally critical issue. World Health Organization (WHO) projects that by 2050, the world's elderly individuals will reach 2.1 billion [1]. WHO has emphasized the benefits of regular physical activities in preventing falls, fall‐related injuries, fragility, and osteoporosis among the elderly, as well as enhancing their physical functions [2]. Physical activity is broadly defined as any movement produced by skeletal muscles that requires energy expenditure [2]. Academic community in the correlation between physical activities and elderly health has been evident since the early 20th century. In 2016, the first bibliometric study in this field was published. It studied papers published between 1980 and 2015, only providing the overall trend and situation about the highly cited publications. However, there appears to be a scarcity of bibliometric analysis and text analysis on this topic within the past 5 years, and the existing one did not discuss global study hotspots, development trends, the cooperative networks, the country centrality, and the citation bursts of this field. Hence, conducting a bibliometric review is necessary to map this field's landscape. This study aims to enable future researchers to understand the academic trends, nationality centrality, collaborative networks, emerging hotspots, and popularity in this field, as well as to identify the core journals, thereby enriching the academic community's comprehension and research of this field.

## 2 Methods

**FIGURE 1:**
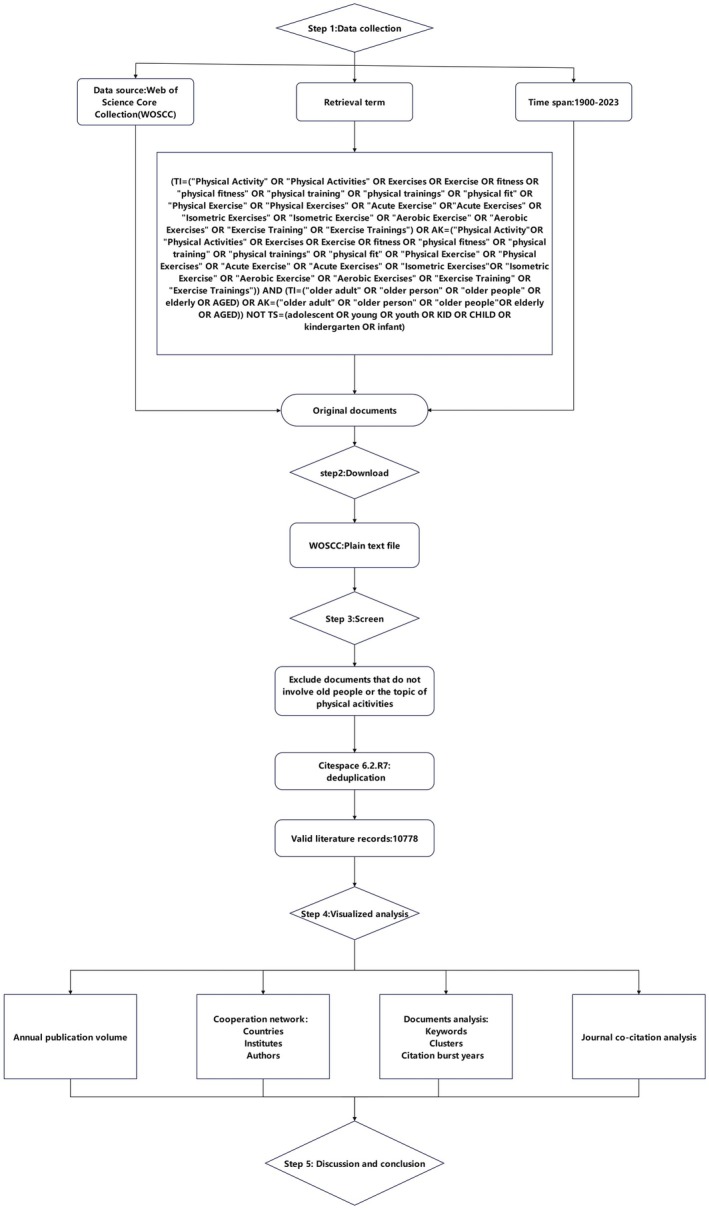
The flowchart of the study design.

The Web of Science Core Collection (WOSCC), developed by Clarivate Analytics, collects papers of sociology, medicine, psychology, and other disciplines. Its collections can date back to 1900. Therefore, it was selected for the literature retrieval. The search formula employed was: (TI = (“Physical Activity” OR “Physical Activities” OR Exercises OR Exercise OR fitness OR “physical fitness” OR “physical training” OR “physical trainings” OR “physical fit” OR “Physical Exercise” OR “Physical Exercises” OR “Acute Exercise” OR “Acute Exercises” OR “Isometric Exercises” OR “Isometric Exercise” OR “Aerobic Exercise” OR “Aerobic Exercises” OR “Exercise Training” OR “Exercise Trainings”) OR AK = (“Physical Activity” OR “Physical Activities” OR Exercises OR Exercise OR fitness OR “physical fitness” OR “physical training” OR “physical trainings” OR “physical fit” OR “Physical Exercise” OR “Physical Exercises” OR “Acute Exercise” OR “Acute Exercises” OR “Isometric Exercises” OR “Isometric Exercise” OR “Aerobic Exercise” OR “Aerobic Exercises” OR “Exercise Training” OR “Exercise Trainings”)) AND (TI = (“older adult” OR “older person” OR “older people” OR elderly OR AGED) OR AK = (“older adult” OR “older person” OR “older people” OR elderly OR AGED)) NOT TS = (adolescent OR young OR youth OR KID OR CHILD OR kindergarten OR infant). The search criteria included articles and review articles published on or before December 31st, 2023. After an initial selection, researchers eliminated documents in which the object is not old people or the topic is not physical activities. After that, Citespace was used to deduplicate the documents. 10,778 publications were included for analysis, providing a large sample size, which is more representative of the entire body of research in this field. The flowchart of the study design is shown as Figure 1. Tools such as Citespace (version 6.4.R1, Chaomei Chen, Drexel University, USA) and Excel (version 2402, Microsoft, Washington State, USA) were used to process and analyze these publications.

## 3 Results

**FIGURE 2:**
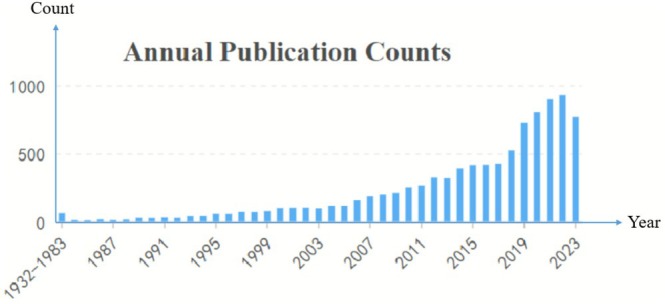
The annual publication counts.

The publications, spanning from 1900 to 2023, are presented in Figure 2. The evolution of this research area can be divided into three distinct phases: the slow development phase, the flourishing phase, and the fluctuating phase. The first relevant publication was retrieved in 1932. From 1932 to 1999, annual publication remained below 100 per year, with a primary focus on the physical health impacts of exercise on the elderly. The period from 2000 to 2022 saw a rapid increase in the annual publications, with research expanding to encompass the mental health and social capabilities of the elderly, and a shift in focus toward the microlevel effects of exercise, such as the impact on estrogen and other microbiological molecules. However, there was a noticeable decline in annual publication, decreasing from 927 in 2022 to 766 in 2023.

**FIGURE 3:**
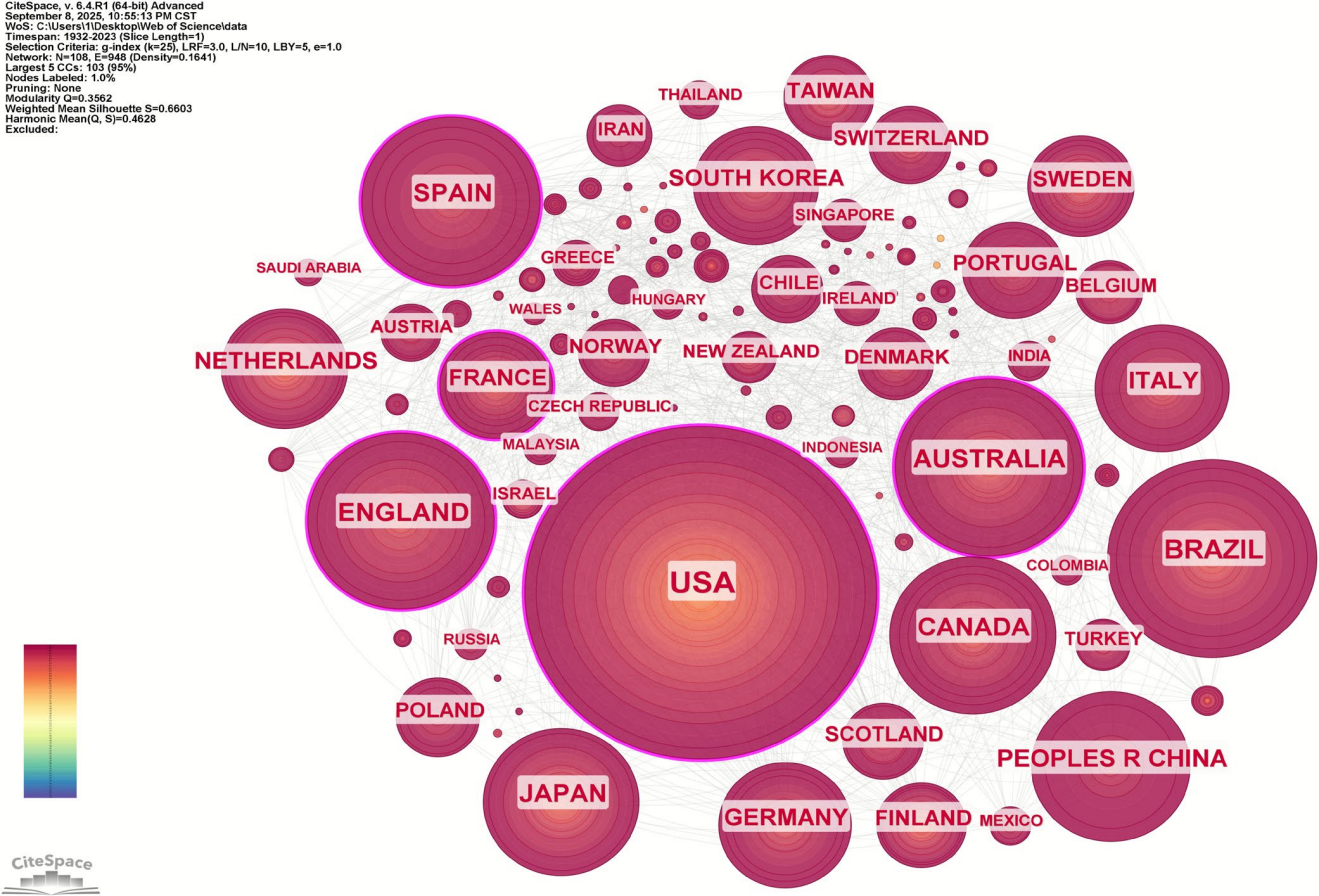
The countries with relatively high centrality.

**FIGURE 4:**
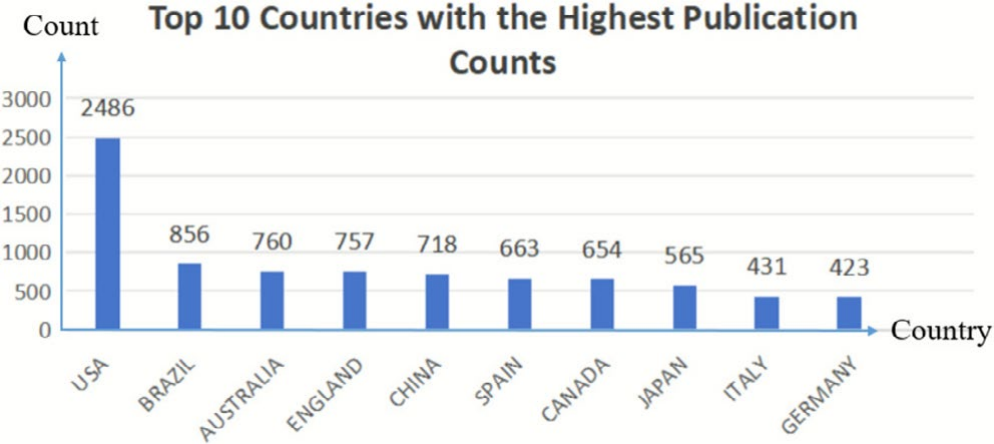
The top 10 countries with the highest publication counts.

In conducting an analysis of the nationality centrality and publication counts using Citespace, it was found that researchers from 107 countries have contributed at least one article each to this field. The countries with relatively high centrality are illustrated in Figure 3, and Figure 4 highlights the top 10 countries with the highest publication counts. A noticeable dominance of Western countries in this domain is observed, likely attributable to their substantial research funding and the high level of expertise of their scientists [3].

**FIGURE 5:**
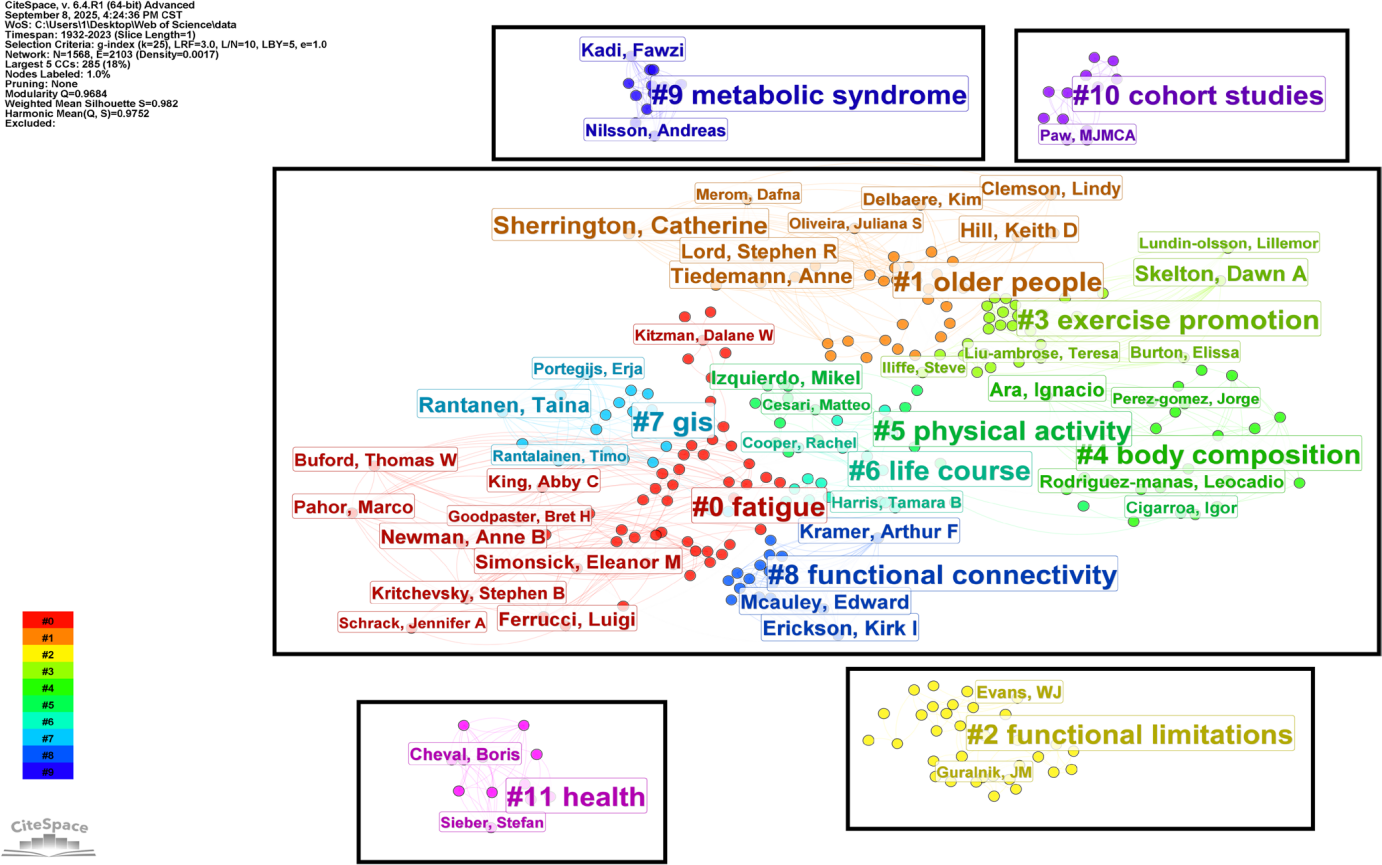
The top 5 largest author cooperation groups.

The study also delves into the intricate web of author collaborations in this area. To date, several significant author collaboration groups have emerged in this field. The top 5 largest groups are depicted in Figure 5. The foremost largest group comprises 8 subgroups, each with distinct research focuses. These focuses broadly fall into two categories: the impact of physical activities on the overall well‐being of the elderly and strategies to encourage exercise among this population. The second largest group primarily investigates the effects of physical activities in relation to metabolic syndrome and sarcopenia in the elderly, especially in conjunction with dietary factors. The third group's research centers on the role of physical activities in mitigating functional limitations in older people. The fourth group focuses on the influence of exercise on the cognitive abilities of the elderly. The fifth group adopts a cohort study method to explore the functional effect of exercise on this age group. Notably, there is a predominant large collaborative group in this field, along with several other groups, with most focusing on the physical impact of exercise on the elderly.

**FIGURE 6:**
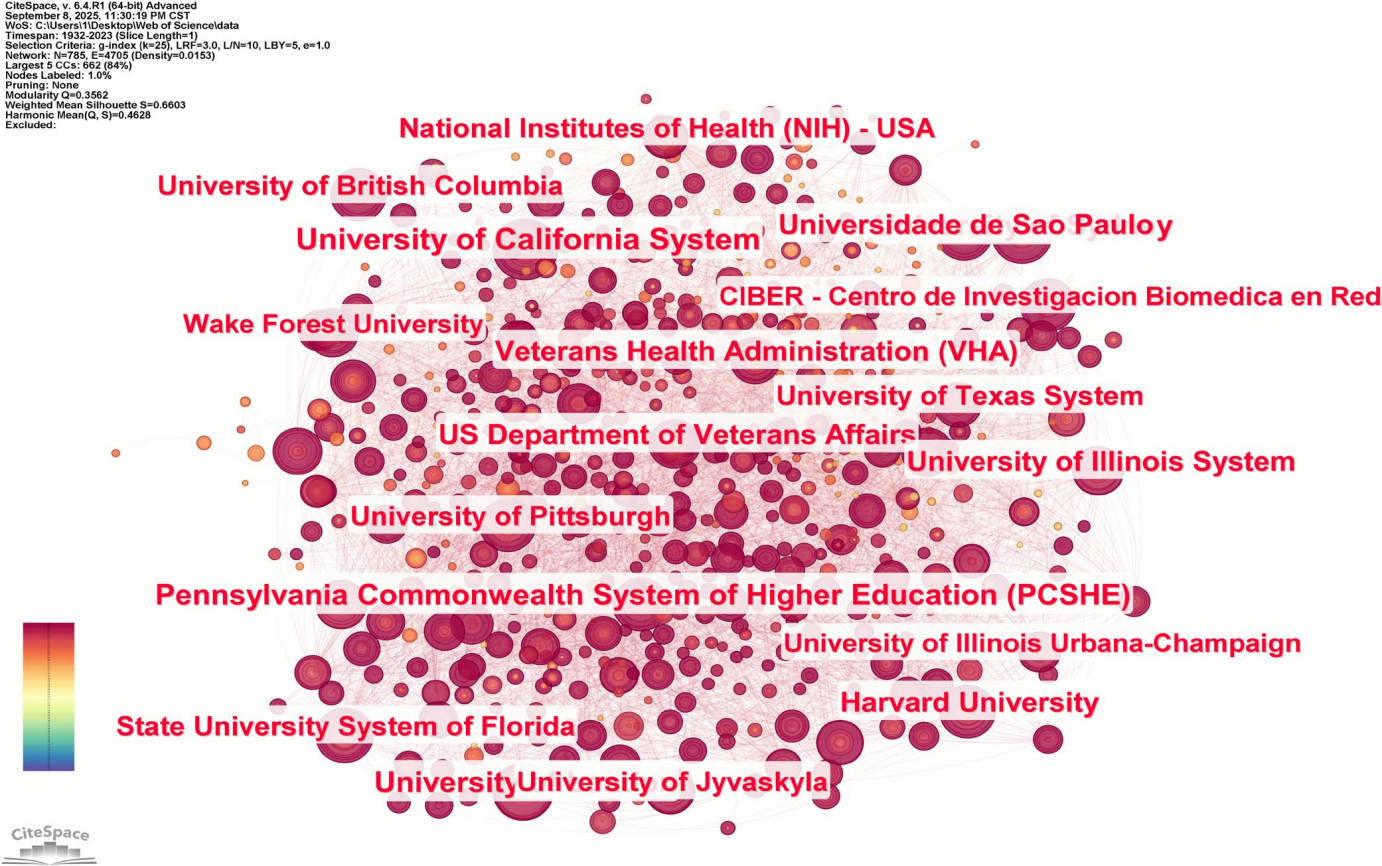
The institutional cooperation networks.

In total, 785 institutions have published at least one article in this area. Utilizing Citespace for analysis, Figure 6 visualizes the cooperation networks among these institutions, highlighting a principal institution group. Collaboration among researchers from these institutions has significantly propelled advancements in this field.

Publications in this field have appeared in 1753 different journals. An analysis of journal citation counts using Citespace was conducted and the top 5 journals with the most citation counts are shown in Table 1. *Medicine & Science in Sports & Exercise* leads with 5009 citations, signifying its status as a leading journal in this field. Besides, journals like *Journals of Gerontology Series A‐Biological Sciences and Medical Sciences*, *Journal of The American Geriatrics Society*, *JAMA‐Journal of The American Medical Association*, and *PLOS ONE* have also amassed over 3000 citations each, underscoring their prominence as core journals in this area.

**TABLE 1:** The top 5 journals with the highest citation counts.

Journal title	Citation counts	Category quartile	JCR category
*Medicine & Science in Sports & Exercise*	5009	Q1	Sport Sciences
*Journal of The American Geriatrics Society*	4305	Q1	Geriatrics & Gerontology
*Journals of Gerontology Series A‐Biological Sciences and Medical Sciences*	4104	Q1	Gerontology
*JAMA‐Journal of The American Medical Association*	3286	Q1	Medicine, General & Internal
*PLOS ONE*	2672	Q2	Multidisciplinary Sciences

**FIGURE 7:**
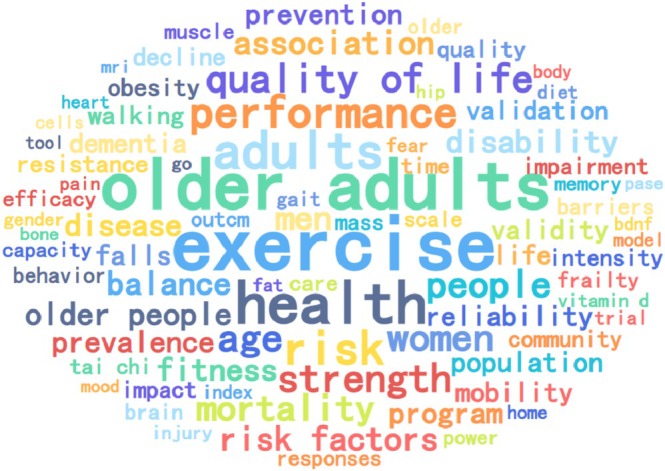
The keyword cloud map.

**FIGURE 8:**
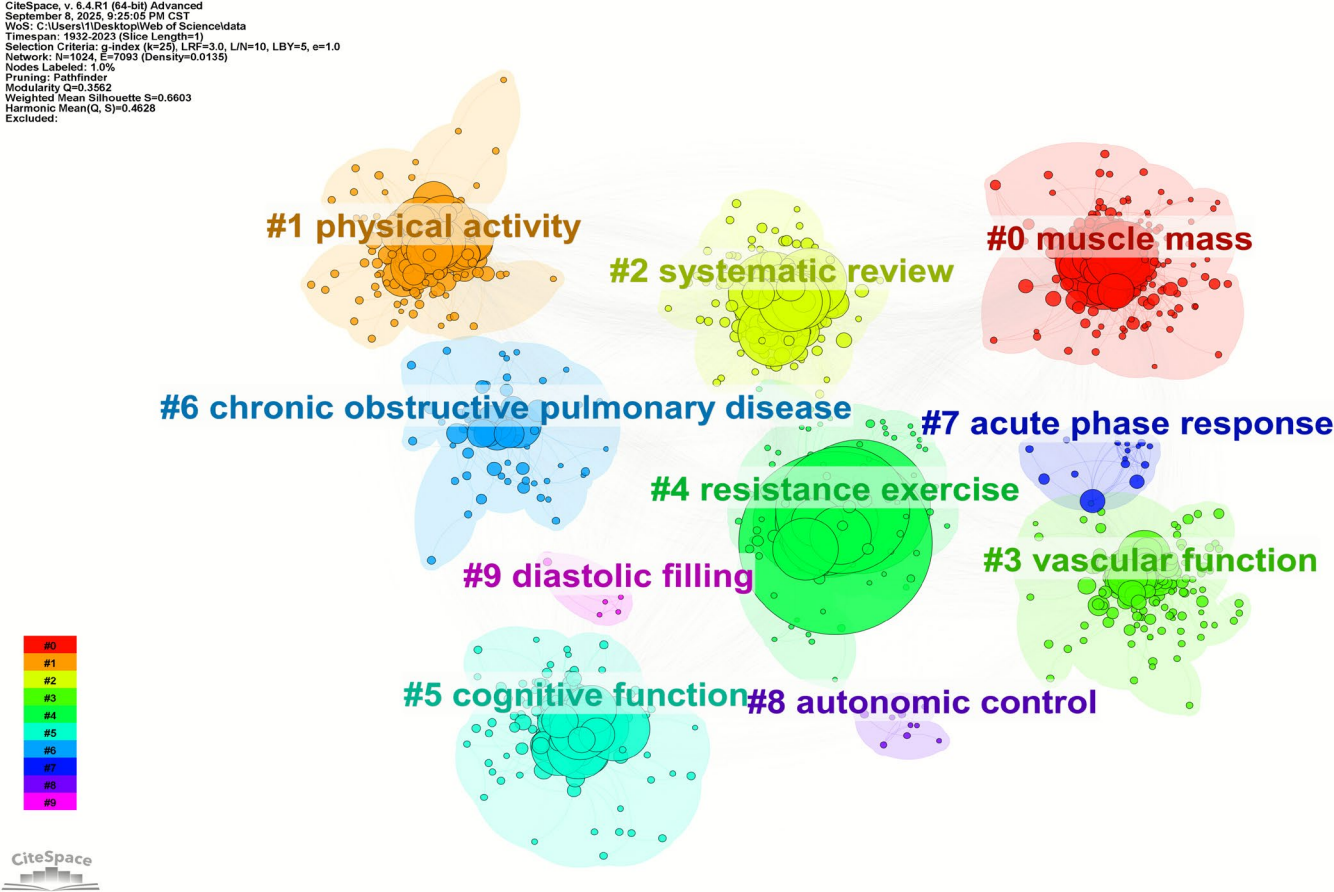
The 10 clusters of the keywords.

We used Citespace to analyze the keywords within the documents we retrieved. The analysis identified 1024 keywords, which were then visualized as a keyword cloud map using WPS, as depicted in Figure 7. Subsequently, these keywords were categorized into 10 clusters as shown in Figure 8. This categorization reveals that the major study domain encompasses both the physical and neuropathic effects of physical activities. Also, we can find that the substantial presence of systematic reviews within these clusters signifies a mature state of research in this area.

Furthermore, we analyzed the citation burst years of the top 25 keywords. This analysis highlighted healthy aging, mental health, and virtual reality as the current trending topics in this area, indicating a shift in research focus toward these emerging areas of interest.

## 4 Discussion

This bibliometric analysis provides a comprehensive examination of the development trends, the latest research foci, and the significant findings within this field. It highlights the close collaboration among researchers, institutions, and countries, leading to the formation of primary cooperation groups. There has been a consistent rise in the annual publication counts. The most recent research trends include healthy aging, the impact of physical activities on the mental health of older people, and the utilization of virtual reality to enhance physical activity in older people. However, the primary contributions are predominantly from developed countries, such as the USA, France, and England. Among the top 10 countries with the highest publication counts, only China, ranked fifth, represents a developing country.

Researchers have identified three key benefits of physical activities for the elderly. First, physical health is bolstered through exercise, a concept studied since the 1930s. Studies demonstrated the benefits from both a microscopical perspective, focusing on the biochemical reactions in cells and tissues, and a macroscopic perspective, concentrating on specific diseases or overall fitness in older adults. Second, mental well‐being is enhanced by physical activities. Increasing recognition of psychological health has led to discoveries linking physical activities with the reduction of mental health disorders, such as depression and post‐traumatic stress disorder [4]. Nowadays, physical activity is increasingly seen as a nonpharmacological intervention to help alleviate loneliness and anxiety in elderly individuals, especially with dementia, while also enhancing their emotional and social capabilities. It is found that physical activity can improve social participation among the elderly, combating loneliness, which is prevalent due to limited familial interaction and social connections. Group exercise, for instance, provides opportunities for social interaction and community building with their peers [5], thereby alleviating loneliness and subsequently improving mental health [6].

The promotion of physical activity among the elderly lies on encouraging participation, prioritizing the frequency and nature of the activity [7]. To support this, governments have implemented various policies, like “Healthy China 2030 Action Plan” [8] and “National Blueprint: Increasing Physical Activity Among Adults Age 50 and Older” [9]. Moreover, advancements in technology and sociology have led to innovative solutions such as the autonomous robotic exercise tutor [10], designed to guide and motivate the elderly to engage in physical activities, while the development of fall monitoring and health monitoring technology also helps prevent falling and detect other acute diseases such as heart attack among the elderly when exercising, enabling them to participate in physical activity more safely [11].

Physical activity is a nonpharmacological way with high benefit–cost to enhance the elderly's physical, mental, and social well‐being. According to WHO, health aging is “developing and maintaining the functional ability that enables well‐being in older age,”, and the functional ability is partly determined by the intrinsic capacity of an individual, which can be improved by physical activity [1]. Hence, in the context of actively promoting health aging worldwide, it is crucial to encourage physical activity among seniors to facilitate the implementation of the UN Decade of Healthy Aging: Plan of Action (2021–2030).

Despite the study's comprehensive scope, it has limitations. First, the articles analyzed are only retrieved from Web of Science. Research published in other databases was not considered. The main proportion of the articles is in English. Some important research in other languages is not considered. Second, some newly published articles have not been cited for many times even though they are important to the development of this area, and “sleeping beauty” phenomenon may exist. However, the Web of Science is a comprehensive database that focuses on academic papers from the interdisciplinary fields of sociology and medicine; therefore, this does not substantially affect the analysis of the scholarly landscape and overall trends.

## 5 Conclusion

This article presents a bibliometric analysis of older adults' health and PA research, addressing a gap in comprehensive bibliometric reviews over the past five years. The steady increase of annual publications indicates the growing importance of the field. Publications were mostly authored by researchers from developed countries in this area. Thus, developed countries appear to lead the study of this topic. China is the only developing country among the top 10 countries with the most publications. However, developing countries account for more than 80% of the global population [12]. Thus, we encourage scholars from developing countries to prioritize research on this topic in the future, to jointly practice healthy aging in their society. The global academic community, especially developed countries, may also support researchers from developing countries in terms of capacity‐building initiatives, funding opportunities, or international collaborations. For example, relative foundations and academic committees should provide more opportunities for researchers from these regions to be included. The academic community focuses on the physical, mental, and social influence of physical activity on older adults. Research focusing on how to assist older adults' exercise with technology to promote healthy aging and on how to translate the research findings into practice is strongly encouraged.

## Author Contributions

Hongman Wang contributed to study design, draft writing, revision and critique of the manuscript, and data analysis. Bo Xie contributed to validation, proofreading, and funding acquisition. Can Liu contributed to the acquisition of part of the data. Yifan Song contributed to participating in proofreading and submitting the manuscript.

## Funding

This study was supported by the Noncommunicable Chronic Diseases–National Science and Technology Major Project (2023ZD0509800).

## Ethics Statement

The authors have nothing to report.

## Conflicts of Interest

The authors declare no conflicts of interest.

## Data Availability

The datasets used and analyzed during the current study are available from the corresponding author on reasonable request.
